# Editorial: Dietary management in kidney diseases: strategies and considerations

**DOI:** 10.3389/fnut.2025.1762006

**Published:** 2026-01-12

**Authors:** Dorin Dragoş, Ruixia Dong

**Affiliations:** 1 Medical Semiology Department, Faculty of General Medicine, Carol Davila University of Medicine and Pharmacy, Bucharest, Romania; 2 College of Horticulture, Jinling Institute of Technology, Nanjing, China

**Keywords:** chronic kidney disease (CKD), dietary restriction, salt intake, protein malnutrition, glomerulopathies, nutrition, volume overload, metabolic acidosis

Kidney diseases, particularly chronic kidney disease (CKD), fundamentally impair the kidneys' ability to rid the organism of the metabolic waste products, the nature and amount of which are determined by dietary intake. The gradual decline of kidney function typical for CKD changes the dietary requirements and restrictions, which progressively gain in importance in preventing the complications and stalling the progression of kidney disease without jeopardizing the nutritional status of the patient (1). The ever more severe dietary restrictions the patient has to abide to include the ratio of plant- to animal-derived foods (particularly as a source of proteins and of acid load), phosphorus and potassium containing foods, salt and water intake, underlining the necessity of tailored dietary adjustments to reflect each patient's clinical status (2).

The aim of this Research Topic was to explore the multifaceted role of dietary management in kidney diseases, including CKD and glomerulopathies. It intended to evaluate how dietary strategies can be optimized in order to: (1) prevent complications such as volume overload, electrolyte disorders (including hyperphosphatemia and hyperkalemia), acid-base disorders (mainly metabolic acidosis), anemia, and bone and mineral disorders; (2) support overall nutritional health (including the avoidance of protein and calorie malnutrition and of sarcopenia); and (3) slow down disease advancement, which may be promoted by diet-related factors including high blood pressure, anemia, urinary protein loss.

Diet is known to be implicated in the etiology of CKD, at least as a key determinant in the cardiovascular kidney metabolic syndrome, being crucially involved in the pathophysiological chain leading to obesity, insulin resistance, type 2 diabetes mellitus, cardiovascular disease, diabetic nephropathy and CKD (3). The role of diet as a whole in CKD was explored by one of the articles published in this Research Topic (Wei et al.). The researchers were able to corroborate the concept that a significant proportion of CKD burden can be diet-related and that this relationship varies across the different regions of the world, concerning trends being noticeable in both low-income as high-income countries. A deficiency in fruits and vegetables is one of the main diet-related factors compounding CKD burden.

Protein restrictions are one of the most controversial issues in the dietary management of patients with CKD (4). One of the articles (Wang et al.) in this Research Topic addressed this issue, particularly the relevance of the plant protein ratio for the outcome of patients with CKD. The authors reached the conclusion that the dietary recommendations regarding the proportion of plant-derived proteins [classified as low (< 33%), medium (33%−66%), and high (≥66%)] should be tailored according to the CKD stage: a medium plant protein intake seemed to be adequate in the early stages of CKD as it correlated with lower mortality as compared with low plant protein diet; however, in the more advanced stages of CKD the benefits of plant-derived proteins are less clear.

Volume overload and high blood pressure are important complications of CKD and both seem to be related to salt intake, which is another controversial issue in the dietary management of CKD patients (5). The article (Shen et al.) in this Research Topic addressing this issue demonstrated that a high sodium intake is an important contributor to the mortality and disability of CKD patients, although there are gender-related and geographic variations.

Metabolic acidosis is another complication that may influence the wellbeing and outcome in patients with CKD and needs to be addressed especially in patients with advanced stages of CKD, one of the strategies consisting in appropriate dietary interventions (6). This is the subject of another article of this Research Topic (Huand et al.). The researchers enrolled 300 CKD patients in a cross-sectional study and divided them into three groups according to dietary acid load (DAL) tertiles. By means of multivariable logistic regression models, the authors found an association between diet-based acid load scores and the combination of CKD and T2DM—this combination was significantly more prevalent in the highest tertile of DAL as compared to the lowest tertile.

The role of diet in more particular kidney disorders was also approached in this Research Topic, one of the articles dealing with the most prevalent among the glomerulopathies: IgA nephropathy (Lu et al.). In a study in which 866 patients with IgAN were recruited and followed for a median of 4.3 years, the researchers demonstrated that vitamin D (VD) supplementation is important in IgAN as it reduces the incidence of major adverse kidney events, hence the conclusion that progressive decline in kidney function is slowed down if optimal levels of VD are maintained on the long term. The reason for this beneficial effect are probably the immune-mediating and inflammation-mitigating effects of VD; in particular, in IgAN VD seems to be involved in modulation of OF NFKB1 and NR4A1 expression in proximal tubular cells.

The role of particular dietary items was also addressed in this Research Topic, namely the general and kidney health promoting actions of parsley (Alobaidi). By systematically reviewing scientific databases, the authors gathered information about the effects of parsley, including diuretic (due to inhibition of the Na+/K + -ATPase and to salt and water excretion promoted by myristicin and apiol), antioxidant (attributed to flavonoids and polyphenols), anti-inflammatory (due to flavonoids and essential oil), antidiabetic (assigned to flavonoids such as apigenin), antimicrobial (attributed to the monoterpenes in the essential oil and to furocoumarins), and many others. The nephroprotective action of parsley appears to be a corollary of the diuretic, antioxidant, and anti-inflammatory effects of parsley. Most evidence consisted of the results of animal studies; only a few small powered human studies were available indicating renal health benefits particularly in obese females.

A very popular topic nowadays is the gut microbiome which seems to be involved in all types of pathological processes, including metabolic syndrome (7), and thereby in cardiovascular kidney metabolic syndrome, a topic tackled by another article (Tian et al.) in this Research Topic. The authors of this article point out that, besides the anti-inflammatory, antioxidant, and lipid improvement effects, gut microbiota also produces short-chain fatty acids (SCFAs) which serve as a source of energy for the human cells, modulate immune and inflammatory processes, fend off oxidative stress, prevent mitochondrial damage, thereby mitigating all the processes responsible for the kidney injury associated with metabolic syndrome.

Hence this Research Topic provides a broad overview of the role of dietary interventions in slowing down the pace of kidney deterioration and ameliorating chronic kidney disease, in preventing its complications (such as metabolic acidosis and protein malnutrition), by both modulating the various pathophysiological processes involved in its progression (particularly inflammation, oxidative stress, immune dysregulation, gut dysbiosis) and preventing/ameliorating systemic disorders involved in the pathogenesis of CKD (especially the metabolic ones: diabetes, obesity, metabolic syndrome).
